# Driving cell response through deep learning, a study in simulated 3D cell cultures

**DOI:** 10.1016/j.heliyon.2024.e29395

**Published:** 2024-04-23

**Authors:** Marilisa Cortesi, Emanuele Giordano

**Affiliations:** a Department of Electrical, Electronic and Information Engineering ”G.Marconi” (DEI), Alma Mater Studiorum – University of Bologna, via dell'Università 50, Cesena, 47521, FC, Italy; b Gynaecological Cancer Research Group, School of Clinical Medicine, University of New South Wales, High Street, Kensington, 2033, NSW, Australia

## Abstract

Computational simulations are becoming increasingly relevant in biomedical research, providing strategies to reproduce experimental results, improve the resolution of *in-vitro* experiments, and predict the system's behavior in untested conditions.

Their use to determine the features associated with an extensive response to treatment and optimize treatment schedules has, however received little attention. To bridge this gap, we propose a deep learning framework capable of reliably classifying simulated time series data and identifying class-defining features. This information will be shown to be useful for the determination of which changes in treatment schedule elicit a more extensive cellular response.

This analysis pipeline will be initially tested on a synthetic dataset created *ad-hoc* to identify its accuracy in identifying the most relevant portion of the signals. Successively this method will be applied to simulations describing the behaviors of populations of cancer cells treated with either one or two drugs in different concentrations.

The proposed method will be shown to be effective in identifying which changes in the treatment protocol lead to a more extensive response to treatment. While lacking direct experimental validation, this result holds great potential for the integration of *in-silico* and *in-vitro* analyses and the effective optimization of experimental conditions in complex experimental setups.

## 1 Background

The *in-vitro* study of cell biology is undergoing relevant developments aimed at increasing accuracy and reliability. These involve both a more widespread use of 3D cell culturing set-ups, to represent more closely the original tissue (e.g. Refs. [[1], [2], [3]]) and innovative non-destructive assays for the quantification of relevant features [[4], [5], [6], [7], [8]].

Within this context computational models have acquired a relevant role being able to infer data not readily available experimentally [9], test hypotheses regarding the molecular mechanisms behind specific macroscopic behaviors [[10], [11], [12], [13]], and predict measurable outcomes in untested conditions [[14], [15], [16]]. Among such tools, suited to be integrated with an experimental analysis, stands SALSA (ScAffoLd SimulAtor), a hybrid continuous/discrete cellular automaton that effectively couples the study of the dynamic evolution of the status and position of the cells, with the representation over time of local changes in resources (oxygen, glucose), drug availability and extracellular matrix's (ECM) mechanical properties [17,18]. While this approach can recapitulate complex experimental behaviors in both tested and untested conditions, it does not provide information on how to modify an experimental protocol to evoke a desired cell response (e.g. to a drug treatment).

To bridge this gap, we here describe an approach for in silico-based optimization of drug treatment schedules in preclinical setups. It combines a deep neural network (DNN) for the classification of temporal series [19], with class activation maps (CAMs) [20] to identify the features associated with a more effective response to treatment. These characteristics will then be exploited to re-design the treatment schedule to improve the response.

Deep neural networks have emerged as universal function approximators [21] and as such have been effectively applied in numerous fields [[22], [23], [24], [25], [26], [27]]. One of the main reasons for their success is the ability to automatically extract complex attributes of the input at a high level of abstraction [28]. This allows for the study and interpretation of complex datasets, such as the ones produced by simulations of biological processes, without any bias or preconceived notion.

CAMs are a method to localize class-identifying features within an image or signal [20,29] and can be used to determine which characteristics contribute to the recognition of each class. These will inform additional simulations aimed at increasing the prevalence of a cell behavior of interest. While the proposed approach is general and applicable to all time-series data, in this work we will focus on the experimental model initially described in Refs. [17,18]. In this computational model, a mesenchymal-like breast cancer cell line (MDA-MB-231) was maintained in a collagen scaffold.

SALSA was demonstrated to be effective in recapitulating the experimentally measured cell proliferation over time, the changes in the mechanical properties of the surrogate extracellular matrix due to the activity of the cells, and the effect of changing the density of the initial population [17]. This simulator could also capture the response of these cells to doxorubicin and mimic an experimentally verified drug resistance mechanism [30] and its proposed resensitization strategy [18]. Indeed, a hypoxic environment was shown by Ref. [30] to trigger the activation of lysil-oxydase (LOX), an enzyme tightly connected with the ability of the cells to remodel their ECM [31], which is also involved in drug penetration and apoptosis inhibition. Treatment with BAPN, a LOX-inhibitor, was shown to reinstate sensitivity to doxorubicin in a dose-dependent manner [18,30]. In our simulations, however, this condition was associated with a higher outcome variability with respect to single-agent treatment [18]. This increase in unpredictability proportional to the set-up complexity, has been also observed experimentally [32] and is currently one of the main limitations in the further development of complex *in-vitro* models.

The method described within this work effectively determines the treatment schedule leading to a more extensive response to doxorubicin. Changing the timing of the administration of the individual drugs yields results comparable with protocols featuring a notably higher dose of the chemotherapeutic agent. This result suggests a potentially viable strategy to reduce exposure to doxorubicin, and thus the severe side effects associated with this drug, without impacting efficacy.

In such evidence resides the importance of computational methods for the study and optimization of complex biological systems. Experimental verification of these results is feasible and encouraged by the free availability of SALSA.

## 2 Materials and methods

### 2.1 Deep neural network (DNN) and class activation maps (CAMs)

**Fig. 1:**
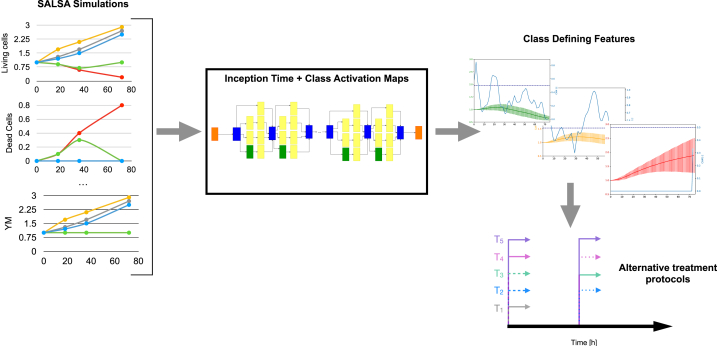
Summary of the workflow of this paper. The results of SALSA simulations, that is the temporal evolution of relevant variables, were used to train InceptionTime to classify treatment outcome. Class activation maps were then used to identify features characteristic of each class and devise alternative treatment protocols.

The DNN used within this work is Inception Time [19], which was specifically developed to classify time series data. Its architecture, outlined in Fig. 1, is inspired by Inception (v4) [33] and as such, it can concurrently extract relevant features at multiple time scales. While the experiments presented in the original work consider univariate time series [19], the method and network structure seamlessly extend to multivariate datasets. Consequently, no modification of the network structure was implemented.

A total of five independent fittings of the classifier were conducted for each dataset (further details in the next section). This strategy, suggested by Ref. [19] can improve the network's performance with respect to a single training step. In all cases, a batch size of 64 and 1500 epochs were used. Accuracy, precision, and recall (more details in the following) were calculated for each trained network and the best performing model was chosen.

CAMs have been computed by projecting back the weights of the output layer onto the convolutional feature maps, as described in Ref. [20] (Equation (1)). Briefly, the result of the last convolutional layer, A(t) is a multivariate time series composed of M variables. Each one of these, *A*_*m*_(*t*) with m ∈ [1, M], results from the application of the *mt*ℎ filter. Filters are the component of the Inception module that enable the extraction of the signal's features at different scales [19]. The CAM for class c can be obtained as the weighted sum of the *A*_*m*_s, where *w*^*c*^_*m*_ is the weight between the output neuron of class c and the *mt*ℎ filter.

(1) $$CAM_{c}\left(t\right)=\Sigma_{m}w_{m}^{c}\cdot A_{m}\left(t\right)$$

in this case, CAMs are univariate time series describing the importance of each time point for the identification of a specific class.

### 2.2 SALSA simulator

SALSA is a computational simulator of 3D cell cultures previously developed by us [17,18]. It couples the description of cell behavior, through a discrete cellular automaton that relies on probabilistic rules to formalize and simulate cell behavior. A continuous cellular automaton describes the diffusion of oxygen, glucose, and pharmacological treatments, beside keeping track of the mechanical properties of the virtual tissue. The continuous variables influence cell behavior (e.g., cells are more likely to double if there are higher levels of oxygen and glucose in their microenvironment), while cells consume resources thus affecting their distribution [17]. The effect of each treatment is modeled using a sigmoid profile that creates a non-linear dependence of cell death on the concentration of the drug [18].

Modifications in SALSA's configuration files enable the definition of different experimental conditions and the simulation of different treatment schedules. SALSA is freely available at https://www.mcbeng.it/en/category/software.html, together with an example of configuration files and a comprehensive user guide.

### 2.3 Datasets

**Fig. 2:**
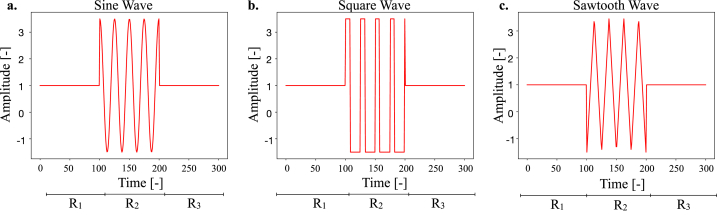
Representative examples of the signals used to test the accuracy of the proposed method.

Within this work, two datasets were used. One of them comprises the SALSA simulations initially presented in Ref. [18], where virtual populations of breast cancer cells with varying degrees of drug resistance (0, 50, 100 % of the population) were treated with doxorubicin (0, 0.03, 0.15, 0.3 ***μ***g/ml) and BAPN (0, 2, 5 mM), alone or in combination. For each of the 1800 simulations, 5 temporal series were extracted. These correspond to the change over time of the number of (i) living or (ii) dead cells, the mean level of (iii) glucose and (iv) oxygen within the scaffold, and (vii) the average matrix Young's modulus. All data were z-normalized and the training set, comprising 70 % of the available data, was composed randomly using an uniform probability distribution. For this dataset, four distinct classes were identified according to the cells' response to treatment [34]. The first one, corresponding to the elimination of the entire population, was called complete response (CR). A decrease of at least 30 % in cell number with respect to the initial population, was classified as partial response (PR), while an increase of over 20 % was identified as progressive neoplastic growth (PNG). Finally, a change in population size between −30 % and +20 % was recognized as stable neoplastic growth (SNG). The other dataset was built *ad-hoc*

to test the localization effectiveness of CAMs for our application. In this regard, signals differing only for a specific region (*R*_2_ in Fig. 2 a.-c.) have been generated and used to train the DNN to distinguish between sine, square, and sawtooth waves. Again, the considered traces were z-normalized, and test (30 % of the data) and training (70 % of the data) sets were composed randomly using an uniform probability distribution.

Table 1 summarizes the characteristics of the datasets used within this work. In all cases the input of the network was provided as ARFF files containing the z-normalized time series and the corresponding labels.

**Table 1:** Summary of the characteristics of the two datasets considered within this work. CR: all cancer cells are killed. PR: the population at the end of the simulation is reduced by at least 30 % with respect to the initial one. PNG: the cell population at the end of the simulation is at least 20 % higher than at the start of the simulation. SNG: the final population size is between −30 % and +20 % of the starting one.

	Signals	SALSA
N. of traces	500	1800
Variables	3 for each signal	5 for each signal
Classes (prevalence)	sine (33 %), square (33 %) and sawtooth (33 %)	CR (0 %), PR (6.2 %), SNG (39.5 %), PNG (54.3 %)
Characteristics	frequency and amplitude chosen randomly between 0.5 and 5 Hz 1–100 (step 0.1 uniform distribution)	extracted from simulations of treatment with doxorubicin (0, 0.03, 0.15, 0.3 ***μ***g/ml) and/or BAPN (0, 2, 5 mM)

### 2.4 Performance metrics and data analysis

The performance of the trained DNNs was evaluated quantifying, for each fitting of the classifier, accuracy (Equation (2)), precision (Equation (3)), and recall (Equation (4)). In all cases, TP, TN, FP, and FN stand for true positives, true negatives, false positives, and false negatives

(2) $$Accuracy=\frac{TP+TN}{TP+TN+FP+FN}$$

(3) $$Precision=\frac{TP}{TP+FP}$$

(4) $$Recall=\frac{TP}{TP+FP}$$

These metrics were computed on the test set, a dataset comprising 30 % of the available time series and not previously used for the identification of the DNN.

After selecting the best network, CAMs were constructed. This analysis, detailed in section 3.1, resulted in a univariate time series for each input signal. As the aim of this work is to identify class-defining features, independently of sample-specific characteristics, all the CAMs belonging to the same class have been averaged and smoothed through a moving mean filter (window size 3 samples).

## 3 Results and discussion

### 3.1 Signal classification and CAMs

To test the efficacy of our pipeline for the identification of class-defining features within multidimensional time series, we analyzed a dataset composed ad-hoc with signals such as the ones in Fig. 2. This choice was guided by the necessity of having a simplified and more controlled benchmark for the validation of the proposed method. All the fitted classifiers were able to correctly and reliably recognize the entire dataset. As such only the first one (iteration 0) will be considered in the following.

After the identification of the DNN, the corresponding CAMs were computed. In this case, we expected the *R*_2_ region to be the most relevant, as it contains the corresponding signal.

**Fig. 3:**
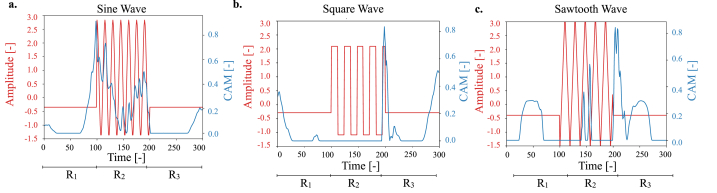
Average CAMs for each class within the signals dataset. Representative time series are shown in red, while the corresponding average CAM is reported in blue.

As shown in Fig. 3 a.-c. our hypothesis is largely verified. CAMs (shown in blue) have low or negligible values in the *R*_1_ and *R*_3_ regions, while high activation is associated with the presence of the actual signal. This result confirms the accuracy of the proposed analysis pipeline for the identification of class-defining features. As such, the same approach was applied to the SALSA dataset.

### 3.2 SALSA simulations classification and CAMs

The results of the fitting procedure on the SALSA dataset are reported in Table 2. A very good performance is achieved by all networks. Since iteration 3 is associated with the highest precision, recall, and accuracy it will be the one considered for the remainder of this analysis.

**Table 2:** Performance measures for the DNN trained on the SALSA dataset. These results are reported independently for each iteration.

Iteration	0	1	2	3	4
Accuracy	0.93	0.93	0.92	0.93	0.91
Precision	0.89	0.90	0.89	0.91	0.87
Recall	0.90	0.91	0.88	0.92	0.90

**Fig. 4:**
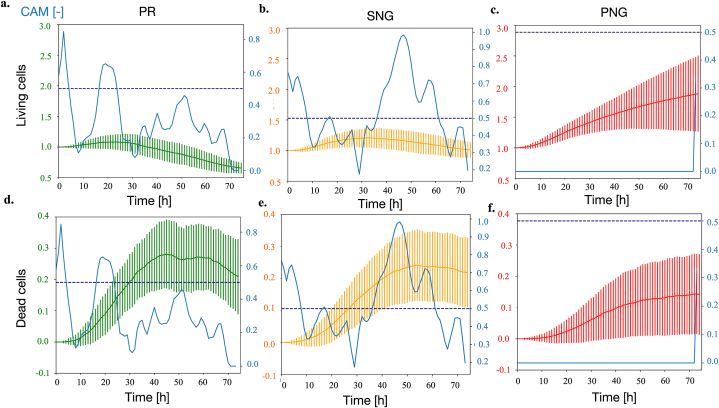
Temporal evolution of the average number of living and dead cells for all classes with prevalence above 0. CAMs (blue trace) and significance threshold (dotted dark blue line) are also reported.

**Fig. 5:**
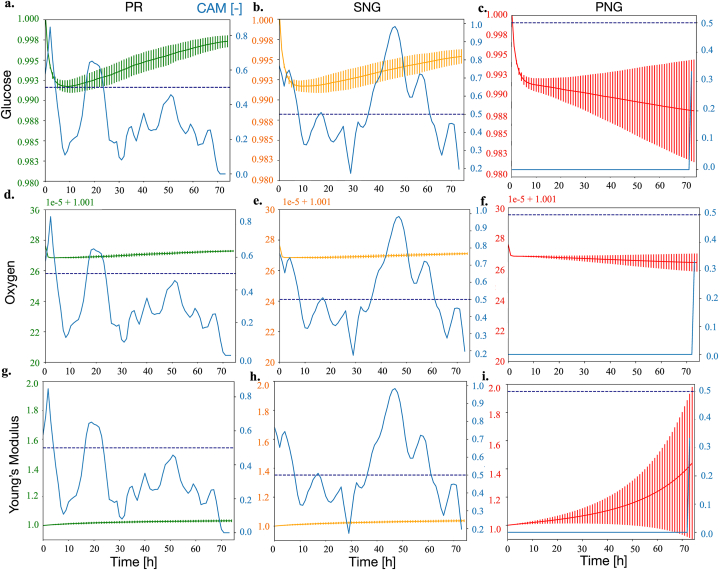
Temporal evolution of the average levels of glucose and oxygen and mean Young's modulus for all classes with prevalence above 0. CAMs (blue trace) and significance threshold (dotted dark blue line) are also reported.

Fig. 4 a.-f. and 5a.-i. report the CAMs for each outcome class with above 0 prevalence (continuous blue lines) overlayed to the corresponding average time series for that class and variable. Additionally, a threshold for relevance of the CAMs values (0.5, dark blue horizontal dotted line in Fig. 4, Fig. 5) will be used to identify the class-defining features. Unlike the signals dataset, the CAMs identified for the SALSA simulations have little overlap. The PNG class, for example, is solely identified by the value of its variables at the end of the simulation, while PR and SNG are mostly characterized by their early and mid-simulation behavior respectively (blue lines in Fig. 4, Fig. 5). Focusing on the regions identified by the CAMs, and comparing the values of the simulated variables for each class, allows to extrapolate class-defining features. These characteristics could provide insights on treatment response in complex settings (combination treatment of chemoresistant cells cultured in 3D) and support with the identification of experimental conditions associated with behaviors of interest (e.g., a more extensive treatment response).

The PNG class is strongly influenced by the steep increase in Young's modulus toward the end of the experiment (Fig. 5 i.). The most extensive response to doxorubicin (PR), on the other hand, was associated (Fig. 4 a., d.) with almost null proliferation and a high rate of cell death at about 20 h after the beginning of the experiment. This behavior was also associated with a more rapid increase in glucose concentration (Fig. 5 a.), that however was determined to be a consequence of the lower cell density, rather than a driver of treatment response. Finally, a rapidly decreasing number of living cells at T = 40 h characterized simulations classified as SNG (Fig. 4 b.).

### 3.3 SALSA simulation of alternative treatment protocols

To test whether the analysis presented in the previous section could support the redesign of the treatment schedule to increase the prevalence of the more desirable PR class, we created four alternative protocols. These were intended to increase cell mortality and reduce proliferation in the interval 17–23 h after the beginning of the experiment.

**Fig. 6:**
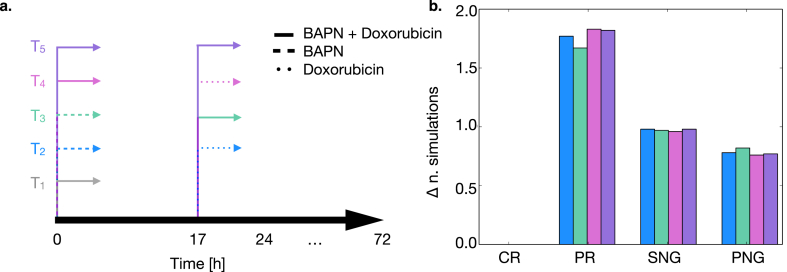
Analysis of alternative pharmacological treatment protocols. a. summary of the tested treatments. Line types identify which drug/drugs were administered at each specific time point. b. results of the application of the 4 alternative treatments in a. (normalized with respect to *T*_1_). Bars represent the change in the number of simulations belonging to each class following the application of each treatment protocol.

Fig. 6a summarizes these alternative schedules and compares them to the treatment protocol used in previous simulations (*T*_1_), where both BAPN and doxorubicin are administered at the beginning of the experiment.

*T*_2_ decouples the administration of the two drugs, adding BAPN at T = 0 h, to induce resensitization to chemotherapy ([30]), and doxorubicin at T = 17 h to promote cell quiescency and death.

*T*_3_ exploits the same principle as *T*_2_ but increases the dosage of BAPN that is administered both at T = 0 h and T = 17h.

*T*_4_, on the other hand, consists in adding BAPN only at T = 0 h, while doxorubicin is administered both at the beginning of the experiment and after 17 h. Finally, *T*_5_ doubles the dose of BAPN and doxorubicin administering them both at T = 0 h and T = 17 h (Fig. 6a).

Fig. 6b shows the percentage change in prevalence for each outcome class when administering the model-informed treatment schedules (*T*_2_-*T*_5_). In all cases, *T*_1_ is used as reference. All alternative treatments result in a decrease in the number of PNG simulations and a corresponding increase in PR ones, while the prevalence of SNGs is substantially the same.

*T*_2_ is particularly effective as it does not increase the amount of drug with respect to the reference *T*_1_, but had a performance akin to *T*_5_, where both doxorubicin and BAPN are administered twice Table 3, Table 4, Table 5 report, for each starting population composition, the breakdown of the prevalence of each outcome class as a function of treatment protocol. When at least a fraction of the initial cells are sensitive to doxorubicin, changing the administration protocol results in the doubling of PR simulations. While totally resistant populations experience an increase of 30 % in partially successful treatments.

**Table 3:** Prevalence of each outcome class as a function of pharmacological treatment for the 0 % drug-resistant-cells population. The percentages for *T*_1_ are different from those reported in Table 1 as for this analysis only treatment with both drugs (at all the different concentrations reported in the methods) was considered.

	PR	SNG	PNG
T_1_	8.33	62.33	29.33
T_2_	16.67	59.33	24.00
T_3_	16.00	57.67	26.33
T_4_	20.67	56.00	23.33
T_5_	17.67	58.33	24.00

**Table 4:** Prevalence of each outcome class as a function of pharmacological treatment for the 50 % drug-resistant-cells population. The percentages for *T*_1_ are different from those reported in Table 1 as for this analysis only treatment with both drugs (at all the different concentrations reported in the methods) was considered.

	PR	SNG	PNG
T_1_	11.00	54.67	34.33
T_2_	23.00	51.67	25.33
T_3_	23.67	52.67	23.67
T_4_	18.33	56.67	25.00
T_5_	22.00	54.33	23.67

**Table 5:** Prevalence of each outcome class as a function of pharmacological treatment for the 100 % drug-resistant-cells population. The percentages for *T*_1_ are different from those reported in Table 1 as for this analysis only treatment with both drugs (at all the different concentrations reported in the methods) was considered.

	PR	SNG	PNG
T_1_	12.33	58.33	29.33
T_2_	16.33	60.67	23.00
T_3_	13.33	60.00	26.67
T_4_	19.00	55.67	25.33
T_5_	17.67	58.67	23.67

The decrease in PNG simulation prevalence, on the other hand, remains approximately the same (about 7 %) in all conditions.

These results underscore the importance of treatment schedule, in outcome determination. While the experimental optimization of this variable is common, the number of conditions and timepoints that are feasible to consider (often 3–5) is too small to enable the comprehensive characterization of its role in treatment response. Computational models and advanced data analysis methods could bridge this gap yielding fundamental insights on treatment response in complex experimental settings while guiding the definition of effective treatment schedules. Indeed, while the modified treatment protocols have been only partially effective in improving response, they could constitute the first step toward a more extensive analysis, where changes in drug dose, timecourse length, and initial population density could be considered.

## 4 Conclusion

In this work, we have shown how a DNN and CAMs canbe used to identify outcome-defining features in simulated *in-vitro* assays. In particular, a more extensive response to pharmacological treatment was determined to be characterized by minimal proliferation and high cell death at about 20 h after the beginning of the experiment. This knowledge, difficult to obtain with traditional mechanistic models, or experimentally, provided useful insights on the studied model (e.g. a favorable outcome is determined very early on) that led to targeted changes in the experimental protocol capable of shifting the outcome distribution. In this regard, a simple 17 h delay in doxorubicin administration resulted in an increase in PR simulations of about 65 %, while avoiding major changes in the prevalence of the SNG class.

These results hold great potential. Indeed they provide an integrated framework for the study of complex 3D cell culture systems capable of extracting key features of the studied system and call for an experimental validation by the preclinical science community. As such they can be used to further our knowledge of the biological mechanisms behind specific behaviors and to aid the definition of new hypotheses to be experimentally tested. They also allow to screening the effect of changing specific conditions through a computational simulator that was shown to consistently replicate experimental data in both tested and untested conditions. The software tools used in this analysis are all freely available online and characterized by high flexibility and thus the ability to adjust to different experimental setups and treatment conditions. SALSA is fully programmable and has been shown effective in replicating multiple experimental setups and treatment conditions [18,35]. DNNs are also ideal, as the same architecture can be trained to effectively classify time series with widely different features, as shown in this and other works [[36], [37], [38]]. As such, while we have focused solely on one cell-culture model and one treatment, the analysis pipeline here presented could serve as a blueprint for other researchers, aiding with the analysis of their complex in-vitro setups and the optimization of the experimental conditions. Training multiple models on the same data, additionally, might lead to the identification of different class defining features, and thus to the availability of more information to drive the design of alternative pharmacological treatments.

Drug development and pre-clinical testing could benefit extensively from this method, providing a strategy to reduce the resources necessary to conduct the analysis and automatically determining the more effective treatment schedules. Indeed, the *T*_2_

protocol identified through the DNN/CAMs analysis outperforms strategies developed by expert users, and its results are equivalent to those obtained when higher cumulative doses of both BAPN and doxorubicin were used.

The automatically-determined time interval between the administration of the considered drugs might however not be compatible with a traditional working schedule. To address this issue and avoid limiting the time points available for the optimization, bioreactors fitted with automatic infusion systems could be employed to conduct the experiment and administer the drugs precisely and accurately as programmed.

Additionally, the use of a bioreactor provides additional benefits, such as increased nutrients diffusion [3,[39], [40], [41]], that further improves the similarities with *in-vivo* biology or the possibility of continuously monitoring the culture [[42], [43], [44]].

Comprehensively, the integration of *in-vitro* and *in-silico* experimentation is becoming a key strategy for the effective characterization of potential therapeutic agents or novel drug combinations. Within this context, the method here proposed holds great potential for the creation of a feedback loop between experimental and simulated results. Indeed, experimental data of appropriate resolution could be used as input for the DNN, instead of the simulated results. The CAMs would thus extract class-defining features for that specific set-up, and suggest modifications that could be tested in-silico prior to their implementation.

## Funding

This document is the results of the research project funded by POR-FESR (2014–2020) Emilia Romagna (PG/2018/632022 DINAMICA).

This project has received funding from the European Union's 10.13039/501100007601Horizon 2020 research and innovation programme under the Marie Skłodowska-Curie grant agreement No 883172”.

## Code and data availability

The code for the simulator is available at https://www.mcbeng.it/en/category/software.html Data available upon request.

## CRediT authorship contribution statement

**Marilisa Cortesi:** Writing – original draft, Visualization, Validation, Software, Methodology, Funding acquisition, Data curation, Conceptualization. **Emanuele Giordano:** Writing – review & editing, Supervision, Conceptualization.

## Declaration of competing interest

The authors declare that they have no known competing financial interests or personal relationships that could have appeared to influence the work reported in this paper.
